# Quality of life in parents of autistic children: A transcultural perspective

**DOI:** 10.3389/fpsyg.2023.1022094

**Published:** 2023-02-23

**Authors:** Valsamma Eapen, Lisa Karlov, James Rufus John, Carmen Beneytez, Poppy Z. Grimes, Ying Qi Kang, Ileana Mardare, Dana Galieta Minca, Laura Voicu, Khasnur Abd Malek, Aishworiya Ramkumar, Krisztina Stefanik, Miklos Gyori, Marta Volgyesi-Molnar

**Affiliations:** ^1^Discipline of Psychiatry, Faculty of Medicine, University of New South Wales, Sydney, NSW, Australia; ^2^Equipo Especifico Alteraciones Graves del Desarrollo, Consejería de Educación de Madrid, Madrid, Spain; ^3^Swalcliffe Park School CIO, Oxfordshire, United Kingdom; ^4^Centre for Clinical Brain Sciences, Division of Psychiatry, University of Edinburgh, Edinburgh, United Kingdom; ^5^Department of Paediatrics, Khoo Teck Puat-National University Children’s Medical Institute (KTP-NUCMI), National University Hospital, Singapore, Singapore; ^6^Department of Paediatrics, Yong Loo Lin School of Medicine, National University of Singapore, Singapore, Singapore; ^7^Carol Davila University of Medicine and Pharmacy Bucharest, Bucharest, Romania; ^8^Department of Primary Care Medicine, Faculty of Medicine, Universiti Teknologi MARA (UiTM), Sungai Buloh, Malaysia; ^9^Institute of Special Needs Education for People with Atypical Behavior and Cognition, ELTE University Budapest, Budapest, Hungary; ^10^HAS-ELTE ‘Autism in Education’ Research Group, Budapest, Hungary; ^11^Institute for the Psychology of Special Needs, ELTE University, Budapest, Hungary

**Keywords:** autism, quality of life, carers, parents, transcultural

## Abstract

**Introduction:**

The concepts of health, illness, and disability as well as the perceptions of autism and quality of life (QoL) vary greatly across cultures and across time. This study sought to explore the interplay of culture on QoL and impact on parents caring for autistic children.

**Methods:**

We used a transcultural dataset from seven countries (Australia, Hungary, Malaysia, Romania, Singapore, Spain, and the United Kingdom) with participating parents/carers reporting on the Quality of Life in Autism (QoLA) questionnaire. The QoLA questionnaire is a validated measure of QoL for parents of autistic children, with Part A subscale measuring parental QoL and part B subscale assessing the parental impact of the child’s autism spectrum disorder (ASD) symptoms or features. We used the Quade’s ranked analysis of covariance to determine significant differences between the countries in relation to QoLA Part A and Part B scores while adjusting for baseline differences using covariates such as parents’ gender, child’s age, and gender. Additionally, a *post-hoc* analysis with Bonferroni correction was also conducted to examine multiple pairwise comparisons.

**Results and conclusion:**

We found that while the effect of features of ASD (Part B subscale) stayed strongly comparable between cultures, the self-reported parental QoL was most likely determined by different aspects of culture in different countries. It is concluded that while the ASD symptoms or features appear to affect parents in the same way across different countries, the parental QoL may be a culturally informed construct.

## 1. Introduction

Autism Spectrum Disorder (ASD) is a life-long neurodevelopmental condition where autistic children and adolescents, or those with ASD generally show differences in a number of areas, mainly characterized by communication or social ability, the presence of restricted repetitive behaviors, and sensory sensitivities (American Psychiatric Association, 2013). The global prevalence rate of ASD is rising with the current estimate at one in 44 children (Maenner et al., 2021). Evidence shows that early detection and support for autistic children is associated with improved outcomes with the American Academy of Pediatrics (AAP) recommending commencing interventions by 2 years of age (Corsello, 2005; Fuller and Kaiser, 2020; Hyman et al., 2020).

Although ASD is a universal disorder with strong biological underpinnings that is diagnosed globally, the symptom presentation, interpretation, reporting as well as treatment strategies appear to be susceptible to cultural influences (de Leeuw et al., 2020; Liu et al., 2022). Culture has been defined as “a set of behavioral norms, meanings, and values or reference points utilized by members of a particular society to construct their unique view of the world, and ascertain their identity” (Alarcón, 2009). Cross-cultural comparisons are critical for better understanding ASD since cultural views regarding appropriate behaviors and normal development for a certain culture may impact parent/carer reports and ultimately influence the timing and nature of ASD diagnosis and treatment (Matson et al., 2017; de Leeuw et al., 2020).

Potential problems with a western-centric way of viewing disability have been consistently raised in the ASD and disability literature. Norbury and Sparks (2013) note that, while ASD is a biologically based condition, it is identified by behavioral markers, and marking the line between normal and abnormal behaviors is a somewhat arbitrary, but a culturally informed exercise. This is illustrated objectively by a study conducted by Carruthers et al. (2018) in which they identified several items on a measure of ASD traits that are universally discriminative in arriving at a diagnosis of ASD, as well as several that discriminated in culture-specific ways, such that they strongly indicated the likely presence of ASD in one country but not in others. An example of this was the item ‘S/he enjoys doing things spontaneously’ which showed clear discrimination between ASD and non-ASD populations in the United Kingdom, but poor discrimination in India and Japan (Carruthers et al., 2018). Beyond these definitional issues lies other important considerations of whether the pathways for assessment, diagnosis, and care that have been successfully built up in developed nations are able to be replicated or made as effective in culturally diverse settings.

In a similar way, stress experienced by parents/carers may be different within different cultures. This is neatly demonstrated by a recent study looking at African American, Hispanic, and White families in the United States, which linked family resilience to parenting stress for parents of autistic children, and showed culture to be a significant moderator of that relationship (Kim et al., 2020; Cheatham and Fernando, 2021). That is, underlying levels of family resilience can influence parental stress differently for parents across different cultures, even when those families are all living in the same geographical location and facing a similar key factor in caring for their autistic children (Kim et al., 2020). Since culture is relevant in informing stress responses, it is anticipated that parental QoL may also be different for people from different cultural backgrounds, even if those people may have similarly defined stressors at play (Durán et al., 2016).

Quality of life has previously been examined with respect to treatment response in an array of cultures. In some cultural contexts, factors such as mindfulness (Lichtlé et al., 2020) and problem-focused coping (Vernhet et al., 2019) have been shown to be helpful for parents of autistic children, while in other cultures, factors including social support, extent of the child’s ASD symptoms, financial wellbeing, parents’ perception of ASD, anxiety about the child’s future, and religion (Ilias et al., 2018) have been identified as important predictors of parents’ wellbeing. Furthermore, the difference in collectivist versus individualist cultures has been highlighted by Kang-Yi et al. (2018), in that, Asian families with a predominantly collectivist culture lean toward traditional forms of support such as religious and community support whereas families from western nations tend to perceive forms of independence, self-reliance, and privacy for their QoL. Therefore, it is reasonable to consider that a parent’s QoL might be influenced by culture. It is also reasonable to consider whether the construct of QoL itself is built differently for people from different cultures.

To address this knowledge gap, the current study aims to answer two broad questions. Firstly, is there an interplay between culture and parents’ perception of how problematic their child’s ASD-specific difficulties are for them? Secondly, is the QoL of parents caring for an autistic child or children, made up of the same or different experiences across different cultures?

## 2. Methods

### 2.1. Participant cohort, collaborators, and selection criteria

Following the publication of Quality of Life in Autism scale (Eapen et al., 2014), several research teams contacted the author about using the measure in their research and a QoLA collaborative network was established of all the users with an offer to collaborate and to make de-identified data available for secondary analysis as relevant/applicable. Seven research groups (Australia, Hungary, Malaysia, Romania, Singapore, Spain, and the United Kingdom) responded to a call out to the network members for a study examining cross-cultural aspects of parental QoL which formed this study group.

Participants were parents or main caregivers of autistic children who were part of a research study using QoLA to examine the parental QoL in seven different countries. The study objectives, place of recruitment, and data collection varied across the seven sites/countries. However, typically, all parents enrolled in the respective studies, for whom, complete QoLA data for Part A and Part B scores were available, were shared in a de-identified way for this secondary analysis. For example, QoLA was administered as part of a school curriculum (United Kingdom cohort), autism early intervention centers (Australia), developmental pediatric clinic within a tertiary academic center (Singapore), or a mix of the above (Spain). The standardized English version of the QoLA was used in Australia, United Kingdom, Singapore, and Malaysia, while local languages were used in the other countries (Spain, Hungary, and Romania). When a non-English version was used, standardization tests were conducted along with a formal process of forward and reverse translation process was followed using the guidelines established by International Test Commission (ITC) for the adaptation of tests from one culture to another. While the QoLA was predominantly administered face-to-face with paper and pen format for completion by parents, some countries (Spain and Romania) also administered QoLA using online forms for completion.

### 2.2. Quality of life in autism questionnaire

The primary measure used was the Quality of Life in Autism (QoLA; Eapen et al., 2014) scale. The QoLA is a QoL measure that has a parent-report version for parents caring for autistic children and adolescents or a self-report version for individuals on the spectrum to complete for themselves. It has two subscales: Part A measures QoL, while part B measures the impact of the child’s ASD symptoms or features. The 28-item Part A comprises questions such as “I am satisfied with my life” or “I feel in control of my life” and was designed to map on to Schalock’s (1996) eight theoretical domains of QoL: emotional wellbeing, interpersonal relationships, material wellbeing, personal development, physical wellbeing, self-determination, social inclusion, and rights. Several items are reverse scored and all are answered on a five-point Likert scale ranging from 1 (Not very much) to 5 (Very much). High scores on Part A, therefore, reflect better QoL.

Part B of the QoLA contains 20-item stems relating to common features of ASD, such as “Sensitivity to certain situations” or “Managing emotional responses.” Here, responses are also on a 5-point Likert scale, but responses range from 5 (Not much of a problem for me) to 1 (Very much of a problem for me). So higher scores on Part B indicate less of a functional impact being experienced in relation to the specific ASD features listed. In this way, Part B acts as an objectively-grounded estimate of the child’s QoL by estimating the extent of the impact on them from their own experience of how ASD is affecting their life. Initial work with the QoLA showed high reliability in both Part A (*α* = 0.94) and Part B (*α* = 0.92), together with sound construct validity (Eapen et al., 2014).

### 2.3. Statistical analysis plan

We used descriptive statistics for continuous variables using mean and standard deviation (SD) and percentages for categorical measures. Overall subscales and their respective domains were tested for normality using the Shapiro–Wilk test. Differences in demographic and QoLA subscales (Part A and B) between countries were assessed by one-way ANOVA with Tukey *post-hoc* test and an independent samples Kruskal-Wallis Test, whereas the Pearson’s Chi-squared tests test for contingency tables was used for categorical data comparison purposes.

Primary analysis was conducted using the Quade’s ranked analysis of covariance to determine significant differences between the countries in relation to QoLA Part A and Part B scores while adjusting for covariates such as parents’ gender, child’s age, and gender. We also conducted *post-hoc* analysis by multiple testing comparisons with Bonferroni correction to examine multiple pairwise comparisons.

Additionally, we used exploratory analysis to determine if the domain-wise ranks of each country were significantly different from their overall rank. The level of significance was set at *p* < 0.05. Further, we also checked the construct and content validity of the subscales in this study, using exploratory factor analysis and confirmatory factory analysis. Additionally, the assessment of internal consistency of both subscales was also assessed using Cronbach’s α. All statistical analyses were conducted using SPSS version 28.0.1.0 (IBM Corp).

## 3. Results

### 3.1. Participant characteristics and descriptive findings

Descriptive characteristics of participants by each country is presented in Table 1. A total of 1,121 parents/carers from seven countries completed the QoLA questionnaire. In terms of the participant characteristics, there were significant differences in parents’ age across the countries, particularly, with a mean difference of 12 years between United Kingdom and Malaysian parents. This is also reflected in their children’s age where children from the United Kingdom cohort were reported to be older than those from the Malaysian sample. However, the gender distribution of parents and children remained similar across the seven countries. The response rate was good to excellent in most of the countries (United Kingdom = 100%; Singapore = 100%; Hungary = 97.7%; Spain = 94.5%; Malaysia = 92.1%; Romania = 92%; and Australia = 68.6%).

**Table 1:** Descriptive characteristics of participants by country.

Variables	Australia (*N* = 96)	Malaysia (*N* = 116)	Romania (*N* = 140)	Spain (*N* = 87)	United Kingdom (*N* = 63)	Singapore (*N* = 97)	Hungary (*N* = 522)	value of *p*
Parent’s age in years, mean (SD)	40.12 (5.93)	35.23 (3.42)	37.76 (6.39)	NA	47.76 (6.94)	38.45 (6.73)	42.55 (8.23)	**<0.001**
Parent’s Gender, count (%)								0.126
Males	9 (9.4)	23 (19.8)	13 (9.3)	9 (10.3)	2 (3.2)	18 (18.6)	80 (15.3)	
Females	79 (82.3)	93 (80.2)	127 (90.7)	78 (89.7)	15 (23.8)	79 (81.4)	440 (84.3)	
Unknown	8 (8.3)	-	-	-	46 (73.0)	-	-	
Child’s age in years, mean (SD)	9.04 (1.57)	6.09 (0.95)	7.02 (3.91)	12.08 (3.70)	15.14 (2.59)	6.22 (1.20)	11.72 (7.86)	**<0.001**
Child’s gender, count (%)								0.080
Males	82 (85.4)	99 (85.3)	106 (75.7)	69 (79.3)	21 (33.3)	78 (80.4)	435 (83.3)	
Females	14 (14.6)	17 (14.7)	34 (24.3)	18 (20.7)	-	19 (19.6)	87 (16.7)	
Unknown	-	-	-	-	42 (66.7)	-	-	
QoLA Part A, mean (SD)	98.31 (21.78)	92.15 (17.50)	90.69 (24.08)	95.57 (20.32)	95.74 (17.93)	103.98 (18.04)	93.35 (19.93)	**<0.001**
QoLA Part B, mean (SD)	71.61 (18.99)	56.57 (12.35)	53.64 (15.79)	53.86 (17.06)	70.61 (16.60)	64.22 (23.06)	48.93 (16.33)	**<0.001**

Bold values indicate statistical significance (*p* < 0.05).

#### 3.1.1. EFA and CFA of Part A subscale

Findings of the EFA showed that Kaiser-Meyer-Olkin (KMO) was 0.94 and the Bartlett’s test was significant (*p* < 0.001), indicating that the data were suitable for factor analysis. Further, the factor analysis of Part A scale of 28 items indicated a five-factor solution with 61.5% of the explained variance. The CFA of the 5-factor model derived from the EFA indicated an acceptable fit of the model to the data, CFI = 0.86, TLI = 0.85, SRMR = 0.05, and RMSEA = 0.08 at *p* < 0.001. Additionally, the internal consistency of the overall Part A subscale was very high (0.95).

#### 3.1.2. EFA and CFA of Part B subscale

For Part B subscale, the Kaiser-Meyer-Olkin (KMO) was 0.95 and the Bartlett’s test was significant (*p* < 0.001), indicating that the data were suitable for factor analysis. Further, the factor analysis of Part B scale of 20 items indicated a three-factor solution with 61.9% of the explained variance. Additionally, the CFA of the three-factor model derived from the EFA indicated an acceptable fit of the model to the data, CFI = 0.91, TLI = 0.89, SRMR = 0.05, and RMSEA = 0.09 at *p* < 0.001. Further, the internal consistency of the overall Part B subscale was very high (0.94).

### 3.2. Quality of life in autism Part A

In terms of Part A subscale on QoL, parents/carers from Singapore reported highest mean QoL scores (mean = 103.9, SD = 22.1) followed by parents/carers in Australia (mean = 98.3, SD = 21.8) indicating greater perceived QoL. Contrarily, Romanian parents/carers reported the lowest mean QoL scores (mean = 90.7, SD = 24.1) indicating least perceived QoL (Table 1).

**Figure 1:**
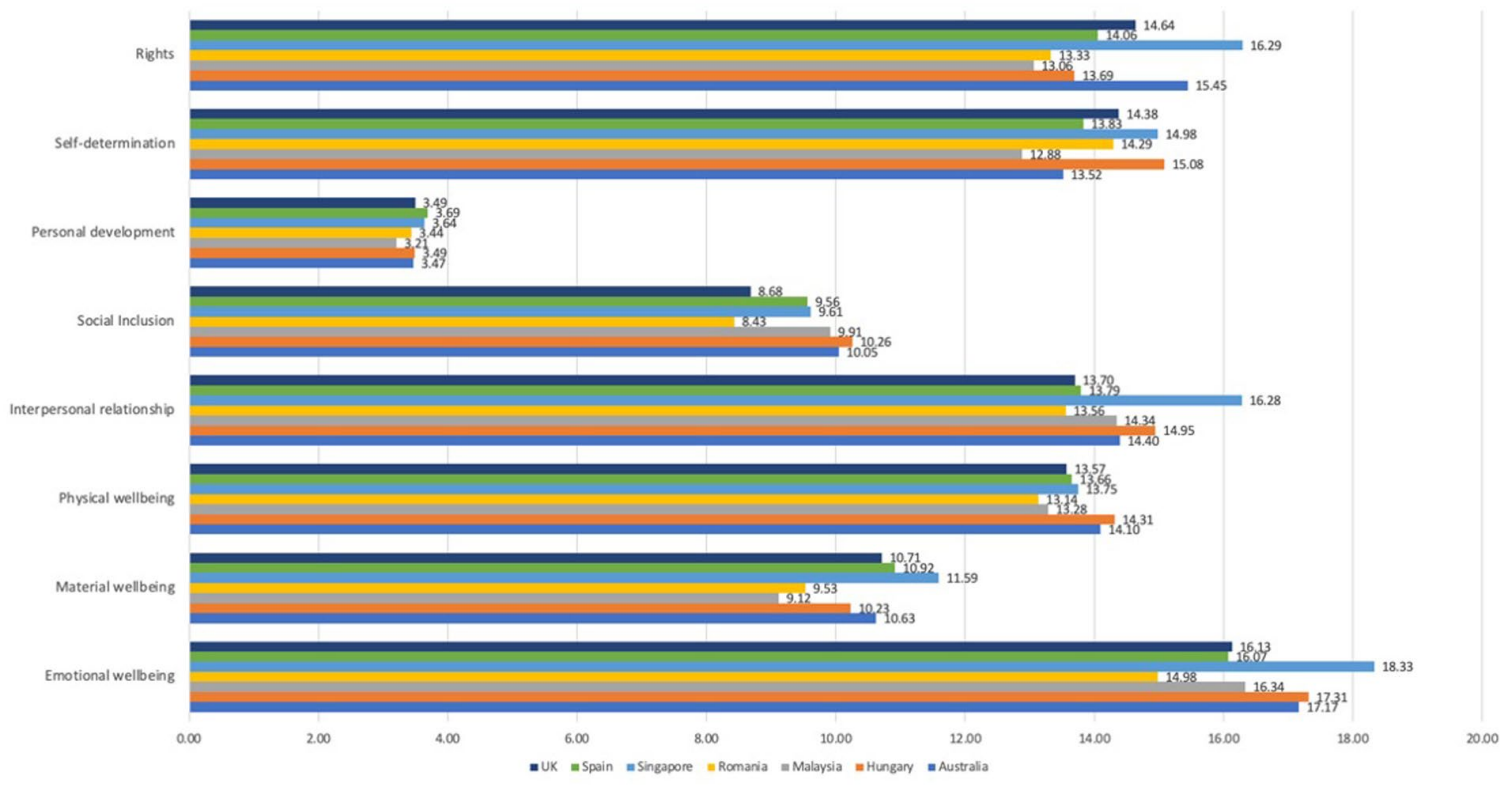
Country-wise comparison of QoLA Part A subscale mean scores by Shalock’s eight domains of QoL.

A country-wise comparison of QoLA subscale A by the eight domains by Schalock (1996) is presented in Figure 1. Here, it can be seen that the top-ranking country, Singapore, had noticeably higher score for the areas of Rights, Interpersonal Relationships, and Emotional Wellbeing. Hungary ranked first in Self-Determination, Physical Wellbeing, and Social Inclusion.

**Figure 2:**
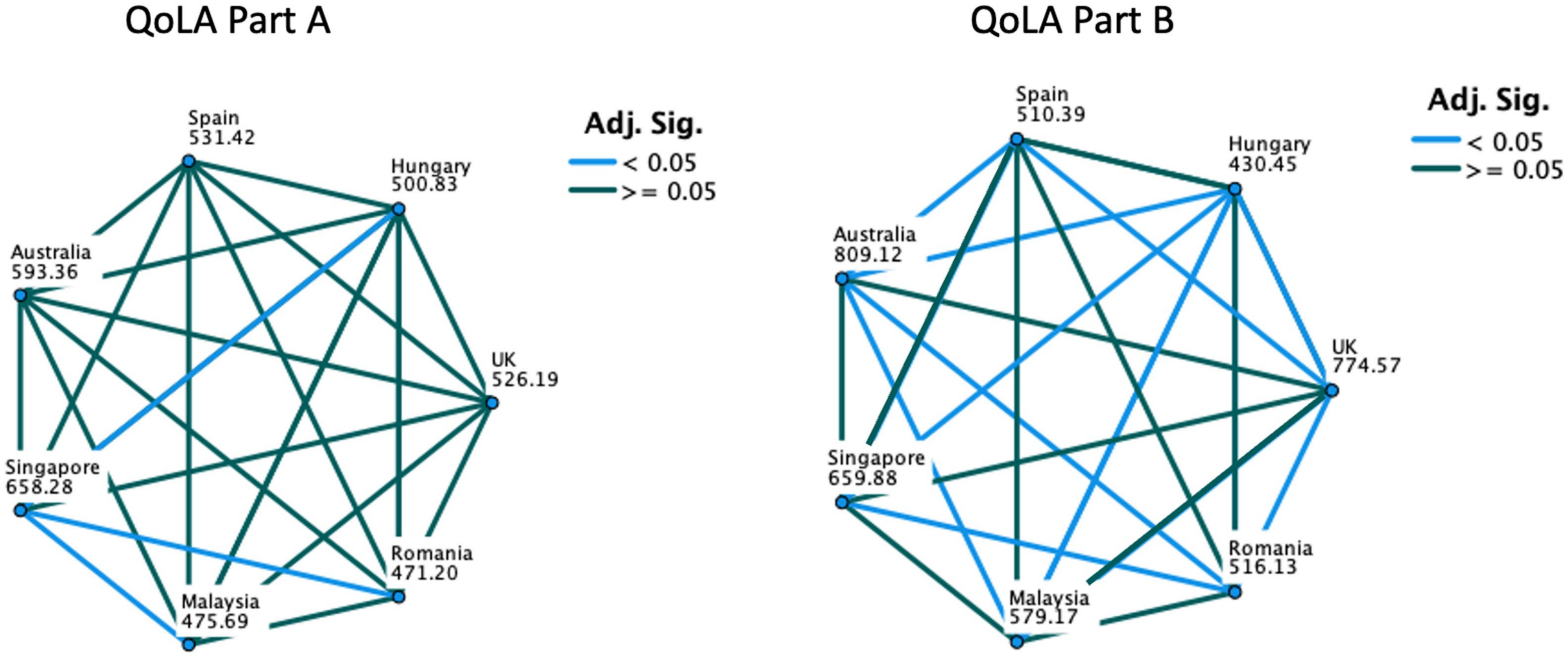
Multiple pairwise comparisons across countries for quality of life in autism (Part A and Part B subscales). Adjusted significance using Bonferroni correction for multiple tests; each node shows the sample average rank of each country for QoLA Part A and Part B subscales.

Primary analysis using the Quade’s ranked analysis of covariance showed significant differences in the mean QoLA Part A scores (F statistic = 4.94, *p* < 0.0001) across the seven countries after adjusting for covariates. While covariates such as child’s age and gender were not statistically significant, parent’s gender was significant. Further, the pairwise comparisons with Bonferroni adjustment showed significant differences in the mean rank scores between Singapore and Romania (mean rank score difference = 174.85 at *p* < 0.001); Singapore and Malaysia (mean rank score difference = 183.64 at *p* < 0.001); and Singapore and Hungary (mean rank score difference = 155.42 at *p* < 0.001; Figure 2; Table 2).

**Table 2:** Multiple pairwise comparisons across countries for QoLA Part A.

Pairwise comparison	Difference	Std. Error	Adj. Sig.^a^
Singapore-Romania	174.85	40.42	0.005
Singapore-Malaysia	183.64	41.95	0.004
Singapore-Hungary	155.42	34.66	0.003

^a^Significance values have been adjusted by the Bonferroni correction for multiple tests. Model adjusted for covariates: child’s age (not significant), child’s gender (not significant), and parent’s gender (significant).

We used an exploratory analysis to determine if the domain-wise (eight domains) ranking of each country were significantly different from their overall rank (overall Part A score). While the majority appeared to show some differences in their rankings in each domain relative to their overall rank, some countries such as Singapore showed very little difference in where they were ranked among the countries in each domain relative to its overall rank. This suggests that the QoL scale is heavily informed by different domains in different countries, and provides a roadmap for future analyzes.

### 3.3. Quality of life in autism Part B

**Figure 3:**
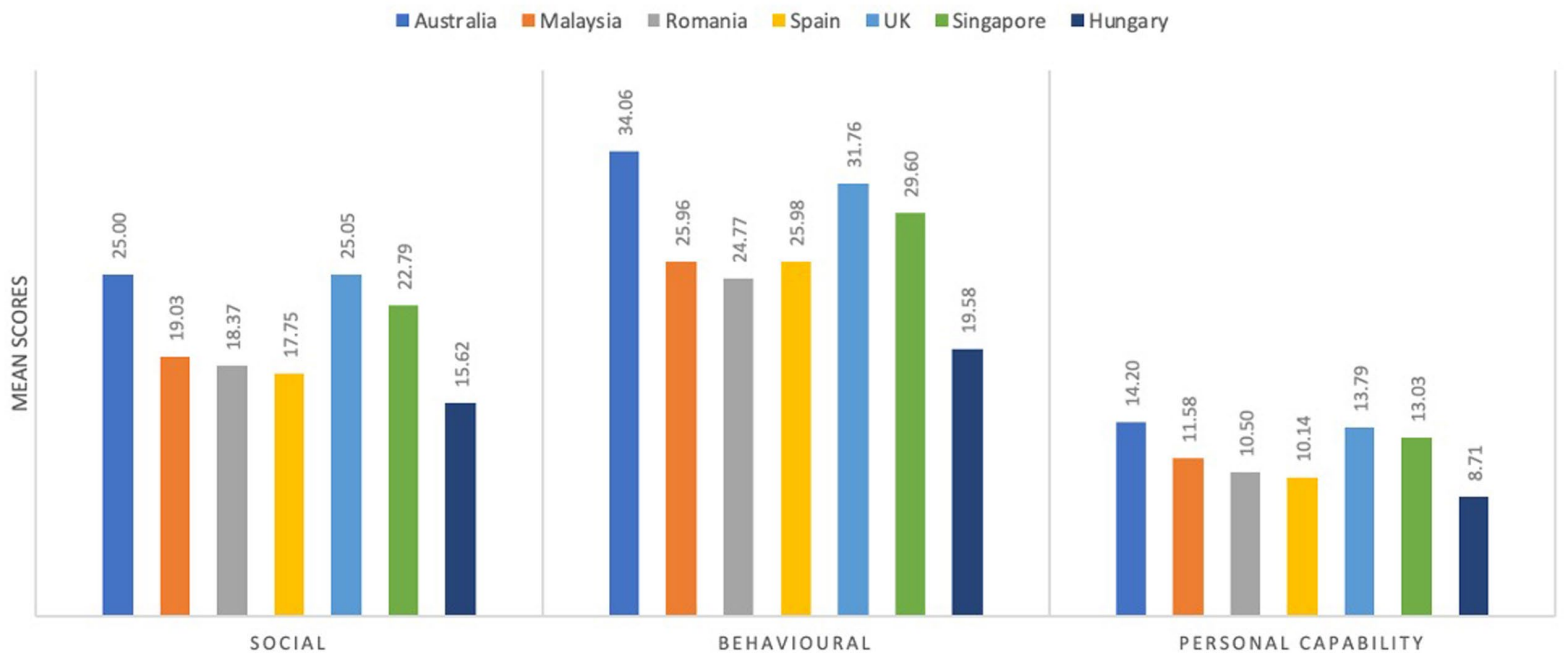
Country-wise comparison of QoLA Part B subscale mean scores by social difficulties, behavioral problems, and personal capability.

For Part B subscale, parents/carers from Australia reported being least impacted by their children’s ASD symptoms (mean = 71.6, SD = 19.0) followed by parents/carers in United Kingdom (mean = 70.6, SD = 16.6) indicating fewer impact from their child’s ASD-related behaviors. Contrarily, parents/carers from Hungary reported lowest scores (mean = 43.9, SD = 17.5) indicating greater problems regarding their child’s ASD-related behaviors (Table 2). Further, we thematically split the 20-item Part B subscale into three major domains: social difficulties, behavioral problems, and personal capability, as summarized in Table 3; Figure 3.

**Table 3:** QoLA Part B items by three thematically derived domains.

Social	Behavioral	Personal capability
Saying things that are socially embarrassing	Showing inappropriate emotional reactions	Doing daily living tasks independently
Understanding others’ feelings	Being overly interested in a particular topic	Managing emotional responses
Responding when approached socially	Engaging in reckless or tactless behaviors	Sensitivity to certain sensations
Understanding the rules of social interaction	Unusual repetitive behaviors or body movements	Communicating needs
Holding a conversation	Taking a literal meaning of comments	
Socializing with people	Destructive behaviors including anger and aggression	
Having friends	Needing to stick to a routine	
	Needing to do things a certain way	
	Getting anxious in a specific situation or during changes	

Findings of the Quade’s ranked analysis of covariance showed significant differences in the mean QoLA Part B scores (*F* statistic = 24.89, *p* < 0.0001) across the countries adjusting for covariates, of which child’s age was statistically significant among other covariates. Further, the pairwise comparisons with Bonferroni adjustment showed significant differences in the mean rank scores across countries (Figure 2; Table 4). In terms of changes to how each country was ranked among the others when looking at overall Part B scores in comparison to the three domains, every country was ranked within one place on the subscales to its overall ranking on Part B. It appears that the differences between countries that were shown on overall Part B scores, then, continued to be shown in the domain-wise rankings of Part B.

**Table 4:** Multiple pairwise comparisons across countries for QoLA Part B.

Pairwise comparison	Difference	Std. Error	Adj. Sig.^a^
Australia-Hungary	370.25	38.23	<0.001
Australia-Malaysia	230.81	44.58	<0.001
Australia-Romania	295.19	43.24	<0.001
Australia-Spain	287.37	47.07	<0.001
Malaysia-Hungary	139.43	29.05	<0.001
Singapore-Hungary	221.24	34.31	<0.001
Singapore-Romania	146.18	39.83	0.037
United Kingdom-Hungary	382.66	69.52	<0.001
United Kingdom-Romania	307.60	72.39	0.006
United Kingdom-Spain	299.79	74.74	0.014

^a^Significance values have been adjusted by the Bonferroni correction for multiple tests. Model adjusted for covariates: child’s age (significant), child’s gender (not significant), and parent’s gender (not significant).

## 4. Discussion

This study investigated the cross-cultural differences in parents’ overall perception of their QoL (Part A) and to assess parents’ perception of how problematic their child’s ASD-specific difficulties or behaviors are for them (Part B), using the QoLA questionnaire. We found that the effect of children’s ASD-specific difficulties on parents (Part B) was strongly comparable between different countries. This pattern shows that the cross-country differences hold at both granular (domain-wise ranks) and overarching (overall score) levels, which, in turn, indicates that the parental perception of their child’s ASD-specific difficulties or behaviors is similar, regardless of which cultural context people come from. Contrarily, we did not find the same pattern for the self-reported parental QoL (Part A) where rankings between countries on each domain differed quite markedly, showing those domains appeared to not be linked to overall scores, such that all rise or fall together. Instead, they appeared to be uncoupled in ways that were meaningful, showing differences that could be seen as speaking to cultural differences in how personal wellbeing in the form of QoL is built.

The Singaporean cohort was ranked highest overall with parents/carers having the most positive QoL in Part A of the QoLA. The high self-rated parental QoL could be related to better accessibility and high quality of healthcare services in the country. In addition, Singapore has a highly subsidized, multidisciplinary early intervention programs catered to autistic children (Ho, 2007; Singapore Government, 2016, 2022; Enable, 2022). Thus, the inherent QoL within the country (Mercer, 2019; The Economist, 2019) in comparison to other countries may also have contributed to the differences observed. Contrarily, parents/carers from Singapore did not rate themselves as being least impacted from their child’s ASD-specific difficulties or behaviors in Part B scale, having only placed as the third lowest level of impact out of the seven countries. The relative weakness of the domain of social inclusion may be related to the significant stigma associated with ASD within the society (Goh et al., 2021). These perceptions may also have contributed to the child’s behaviors being viewed as having a significant impact by parents and the high Part B scores.

The Australian cohort of parents/carers rated being least impacted from their child’s ASD-specific difficulties or behaviors (Part B), and the second highest parental QoL (Part A). However, there was some variation in the domain-wise findings in Part A, indicating some underperformance for self-determination and personal development, with the main areas of relative strength being physical wellbeing and social inclusion. The sample from the United Kingdom was ranked similarly to Australia, ranking third for QoL and second for positive rating of Part B. However, in contrast to the Australian sample, the balance among the domains in the sample from the United Kingdom indicated higher than expected performance in material wellbeing, personal development, self-determination, and rights. The high ranking of both these counties could again be explained by better accessibility and high quality of mental health services, particularly, dedicated early supports for ASD compared to other nations (Australian Institute of Health and Welfare, 2016; United Kingdom National Health Service, 2021).

The Hungarian sample did not rank highly overall, but performed quite strongly on self-determination and physical wellbeing domains in Part A. These deviations in rank order could be indicative of some overall cultural differences. Similarly, overall QoL in Hungary (Kopp and Kovács, 2006) and Romania is generally self-rated as being unsatisfactory, with a combination of individualist and survival values (David, 2015; World Values Survey, 2020), meaning that people strive and achieve their own improvements to their lives without relying on a system to provide for them.

Parents/carers from Malaysia self-rated second lowest overall level of QoL, but strongly rated in some domains such as social inclusion, emotional wellbeing, and interpersonal relationships. It ranked fourth out of the seven countries in terms of the rated severity of impact of autism features. Data from Malaysia sampled mainly mothers. Within the local culture-specific role for mothers, household duties typically involve household chores and caring for the children (Hirschman, 2016). Mothers in this study may accept and adapt to their situation (Ilias et al., 2017) and continue performing their “role” to take care of the children in the best possible way supported by the extended family and other social, faith, and community supports. This, in turn, may explain the relative better performance on social inclusion, emotional wellbeing, and interpersonal relationships.

Finally, the sample from Spain showed the most similar ranking in both Part A and Part B scales, ranking at the same position among the other countries. In terms of the domains, the Spanish cohort had positive ratings for personal development and material wellbeing, but ranked lower in emotional wellbeing. The Spanish sample collected data on parental stress and depression (data not shown for consistency with other countries) which showed that 78.1% of parents reported feeling stress and 60.9% feeling depressed or anxious. These results are in consistent with previous Spanish studies that have found significant association between presence of clinically significant stress and parental QoL (Cabanillas et al., 2006; Durán et al., 2016).

This study has several strengths and limitations. A major strength of the current work was the fact that a multinational collaboration was built, thus enabling a novel exploration of QoL in parents of autistic children, through a transcultural lens. There are a number of limitations to consider when interpreting the findings. Firstly, data have been collected in different ways for different purposes and with different sampling procedures within each contributing country. While we have taken some measures to statistically control for different sampling, we cannot rule out the possibility that differences in factors such as ASD severity, socioeconomic status, and parental stress across the countries may result from procedural and methodological differences across sites. Additionally, some key variables (as mentioned above) were not collected at all sites; hence, we were unable to use in the analysis and for robust comparison using matching techniques. Alternatively, we have accounted for the baseline differences by using statistical analysis such as Quade’s ranked analysis of covariance to adjust for key covariates such as parents’ gender, child’s age, and gender. Therefore, future research should aim to organize a transcultural research collaboration prior to data collection in order to maximize the collection of comparable information at different sites. This will render making more accurate comparisons and share findings with greater ease.

It is concluded that while features of ASD and their effect on people’s lives are judged similarly by people from different cultures, parental QoL is influenced, to some extent by the culture to which the parent belongs. The findings of this research should be considered preliminary and highlight an area of need in ASD research. Enlarging this vision of this research and achieving a better understanding of ASD internationally has the potential to provide a multitude of benefits. Directions for future research should focus on investigation of specific sociocultural factors that impact parental QoL and perception of their child’s ASD-specific difficulties or behaviors. Further explanations of these findings will be not only illuminating for how people from different cultures define themselves and their circumstances, but also for considering culturally appropriate supports for parents/carers who undertake a significant caring role within their families.

## Data availability statement

The original contributions presented in the study are included in the article/Supplementary materials, further inquiries can be directed to the corresponding author.

## Ethics statement

The studies involving human participants were reviewed and approved by for Malaysia: Research Ethics Committee, Research Management Institute, Universiti Teknologi MARA (UiTM; 600-IRMI) (5/1/6). For Australia: University of New South Wales Human Research Ethics Committee (Project No: HC14267). For Spain: Doctoral commission Faculty of Education, Universidad Complutense de Madrid. For Hungary: Committee of Research Ethics of Bárczi Gusztáv Faculty of Special Education, Eötvös Loránd University, permission number: KEB/2017/003. For Singapore: National Healthcare Group, Domain Specific Research Board, Singapore. For United Kingdom: UCL research group (CRAE). For Romania: University Ethics and Deontology Commission of “Carol Davila” University of Medicine and Pharmacy Bucharest. Written informed consent to participate in this study was provided by the participants’ legal guardian/next of kin.

## Author contributions

VE contributed to conceptualization of the work, leading data collection at one site, and to writing the article. LK contributed to conceptualization, analysis, managing the collaboration, and writing, including writing the first draft of the article. JJ conducted the majority of the analyzes and contributed to the writing of subsequent drafts. CB, PG, YK, IM, DM, LV, KM, AR, KS, MG, and MV-M each contributed to data collection at their site and to the writing of subsequent drafts of this article. All authors contributed to the article and approved the submitted version.

## Funding

Research at one site was supported *via* a research grant from the Hungarian Academy of Sciences within its Content Pedagogy Research Program (2016–2021 period).

## Conflict of interest

The authors declare that the research was conducted in the absence of any commercial or financial relationships that could be construed as a potential conflict of interest.

## Publisher’s note

All claims expressed in this article are solely those of the authors and do not necessarily represent those of their affiliated organizations, or those of the publisher, the editors and the reviewers. Any product that may be evaluated in this article, or claim that may be made by its manufacturer, is not guaranteed or endorsed by the publisher.
